# Establishment and characterization of organoids from a patient with adenomyoepithelioma of the breast

**DOI:** 10.1080/21655979.2021.1974809

**Published:** 2021-12-07

**Authors:** XiangRong Luo, JianTao She, Tao Xu, Yuan Zhou, ChuanBo Xu, JianPing Jiang, TianGang Li, Huajiang Liu, Hui Shen, Bolong Yin, Bin Dai

**Affiliations:** Department of Breast and Thoracic Surgery, The Central Hospital of Shaoyang, Shaoyang, China

**Keywords:** Adenomyoepithelioma of the breast, organoid culture, drug sensitivity test, breast cancer, 3-d breast cancer organoids

## Abstract

Adenomyoepithelioma (AME) of the breast is a rare tumor that is composed of proliferating epithelial and myoepithelial cells. The pathogenesis of AME remains unclear, and no breast cancer cells have been identified in such tumor tissues. In this study, we established patient-derived breast cancer organoids from the surgical tumor samples of an elderly Chinese woman with an AME of the breast. Our findings confirmed the successful establishment of organoids from an AME of the breast of this patient. A short tandem repeat analysis revealed that the DNA signature of the AME of the breast organoids matched the DNA signature of the original tumor specimen. Moreover, diameter assay confirmed that the organoids from the breast AME showed sensitivity to paclitaxel and doxorubicin treatments, which was similar to, but lesser than that of primary culture cells. In conclusion, we established an efficient 3-dimensional breast cancer organoid culture platform from an AME of the breast. This platform can be effectively used for exploring clinicopathological and genomic characteristics of AME of the breast to identify possible treatments and increase awareness about this disease entity.

## Introduction

Adenomyoepithelioma (AME) of the breast is a rare tumor composed of benign or malignant epithelial and myoepithelial cells. In the 1970s, Hamperl introduced the concept of AME for the first time as a tumor consisting of ducts/lumen and myoepithelial cells [1]. In 2012, the World Health Organization defined AME as a tumor formed by the proliferation of myoepithelial cells surrounding the small lacunae overlying glandular epithelium [2]. Only few AMEs undergo malignant transformation with local recurrences or distant metastases [3]. It is difficult to differentiate benign AME from malignant AME, which is characterized by increased nuclear fission, cell heterogeneity, necrosis, coarse chromatin, cell-invasive growth, satellite foci, prominent nucleoli, and an increased mitotic rate [4–6].

Almost all prior studies on AME used clinical samples. Most of the AMEs possess gene-specific heterogeneity and display recurrent mutually exclusive mutations of AKT serine/threonine kinase 1 (AKT1) and phosphatidylinositol-4,5-bisphosphate 3-kinase catalytic subunit alpha (PIK3CA). Thus, PIK3CA and AKT1 may serve as effective therapeutic targets of AME in clinical settings [3]. Moreover, MYB gene rearrangements do not appear in AMEs, but they frequently appear in adenoid cystic carcinoma [4]. However, the molecular characteristics of AME remain largely unknown owing to the lack of an ideal research model. Therefore, the establishment of a feasible and robust tool for further investigation on the pathogenesis of AME is warranted.

The concept of three-dimensional (3D) organoid culture has garnered immense attention recently. An organoid culture system includes bioengineered microenvironments that drive cells to self-organize into structures that mimic human tissues and organs from which they were derived [7,8]. Organoid cultures have been established from various primary tumor tissues, including cervical [9], colorectal [10], lung [11], liver [12], gastrointestinal [13], prostate [14], bladder [15], and pancreatic [16] cancers. Human mammary epithelial organoids have been established as a useful preclinical model because the histology, transcriptome, and genome of a breast cancer organoid typically matched the original tumor [17]. However, organoids derived from patients with an AME have not been reported so far.

Therefore, we thought the case of a 68-year-old Chinese woman with an AME of the breast can be constructed as organoids. This study attempted to establish an AME organoid system from original AME samples to study the drug targets and drug sensitivity of AME. Results showed that we established an AME organoid model, and the organoids were found to be sensitive to the chemotherapy drugs paclitaxel and doxorubicin. To the best of our knowledge, this is the first time that AME patient-derived breast cancer organoids have been established.

## Methods

### AME sample collection

At the time of surgery, the AME tissue was collected from the patient with an AME of the breast. The AME sample was washed three times with precooled phosphate-buffered saline or saline to eliminate congestion. Soybean-size tissues were obtained from the AME sample and put into cryopreservation tubes (Orgen biotechnology, Guangzhou, China) containing tissue protection liquid within 30 minutes and stored at −4°C temporarily. This experiment was approved by the committee of the Central Hospital of Shaoyang, and informed consent was obtained from the patient.

### Immunohistochemistry assay

Briefly, the deparaffinized sections were incubated in citrate buffer solution (Nanjing Jiancheng Bioengineering Institute, Nanjing, China) for antigen retrieval. Subsequently, 3% hydrogen peroxide ((Nanjing Jiancheng Bioengineering Institute) was used for blocking endogenous peroxidases. Slides were then incubated with primary antibodies at 4°C overnight, followed by incubation with secondary antibodies at 37°C for 30 min. Finally, 3,3-diaminobenzidine was used for staining and hematoxylin was used for counterstaining the nuclei. The primary antibodies were as follows: Vimentin Polyclonal Antibody (10,366-1-AP; Proteinte ch, Wuhan, China), Pan-Keratin monoclonal mouse antibody (4545 T; Cell Signaling Technology, Danvers, MA, USA).

### Organoids and tumor cell culture

Soybean-size tissues were cut into 1 mm^3^ pieces and washed with 3 mL Hanks’ balanced salt solution (HBSS) (14,025,092; Gibco, Grand Island, NY, USA) containing 1% penicillin/streptomycin (15,140,122, Gibco). The tissue pieces were then digested in 2 mL HBSS containing 1% penicillin/streptomycin and tissue digestive fluid (Orgen biotechnology) in a 37°C water bath for 30 minutes. During this period, the digested tissue was shaken once every 5 minutes. Digested cells were passed through 100 µM cell filters to remove excess tissue. The collected cells were suspended in 4 mL HBSS, followed by centrifugation at 1200 rpm. The collected cells were then suspended in a precooled AdDF+++ medium (advanced DMEM/F12 medium [12,634,010, Gibco] containing 10 mM HEPES [15,630,106, Invitrogen, Carlsbad, CA, USA], 1 × GlutaMAX [35,050,079, Gibco], recombinant human R-Spondin-1 [7189–10, Biovision, Guangzhou, China], Noggin [HEOPP-1403, Cyagen, Guangzhou, China], EGF [RP-8661, Invitrogen], B27 [17,504,001, Invitrogen], N-Acetylcysteine [HY-B0215, MedChemExpress, Shanghai, China], 500 nM A83-01 [2939, APExBIO, Beijing, China], 5 μM Y-27632 [B1293, APExBIO], and 1% penicillin/streptomycin). Subsequently, 1 × 10^5^ cells per well were seeded into a preheated 48-well plate. BD Matrigel™ basement membrane matrix (354,234, BD Biosciences, San Jose, CA, USA) was thawed on ice. A mixture of 25 µL Matrigel and 25 µL cell suspension was seeded into a 96-well plate, which was incubated at 37°C in a humid environment with 5% CO_2_ for 30 minutes. Subsequently, 100 µL AdDF+++ was added to each well. The medium was changed every 2 days.

### Short tandem repeat identification

Short tandem repeat (STR) identification was conducted by Cellcook Biotech Co., Ltd (Cellcook, Guangzhou, China). The genomic DNA of original AME samples and organoids was isolated using a DNA isolation kit (TianGen Biotech, Beijing, China). Subsequently, the genomic DNA was dissolved in sterile deionized water, and 1 ng of the DNA was used for each polymerase chain reaction (PCR). The reagents for PCR included DNA template, 2× PCR buffer, 1.5 mM MgCl_2_, 0.4 mM each dNTP, 0.1 mM STR site primers, and 0.25 UmL^−1^ Taq DNA polymerase, and water. The total reaction volume was 50 μL. The PCR products were used to perform capillary electrophoresis. Results were analyzed using the ‘Gene Marker’ software.

### Drug sensitivity test

Primary AME-derived tumor cells were seeded into a 96-well plate at a concentration of 1 × 10^4^ cells/well. The following day, the cells were exposed to different doses of paclitaxel or doxorubicin for 48 h, which time was selected following with previous study [18,19]. Subsequently, we added Cell Counting Kit-8 (CCK-8) solutions (10 μL) (HY-K0301, MedChemExpress) to each well and incubated the plate for 2–4 h at 37°C. We determined absorbance values at 450 nm using an automated microplate reader (Multiskan FC, Thermo Fisher, Waltham, MA, USA).

When the diameter of AME organoids was approximately 50 μM, they were exposed to different concentrations of paclitaxel or doxorubicin for 48 h. The morphology and size of the AME organoids were then observed using an optical microscope.

## Statistical analysis

Statistical analyses were conducted using GraphPad Prism software (8.0; La Jolla, CA, USA). All experiments were repeated thrice and the data in this study are presented as mean ± standard deviation. Differences between groups were compared using one-way ANOVA and analyzed using SPASS 18.0 (SPASS, Chicago, IL, USA), followed by Duncan’s post hoc test. *P*-values of <0.05 were considered statistically significant.

## Results

We encountered a patient with AME of the breast, which is rarely observed in clinical settings. To establish an AME research model, we created AME organoids. STR identification was used to compare the DNA of the organoids and the original tissues to check for similarity between the DNA signatures of the two. The chemosensitivity of AME organoids was investigated using the chemotherapeutic drugs paclitaxel and adriamycin. The results showed that we established an efficient 3D breast cancer organoid culture platform from an AME of the breast. This platform can be effectively used for exploring clinicopathological and genomic characteristics of AME of the breast to identify possible treatments and increase awareness about this disease entity.

### Case report

A 68-year-old Chinese woman was admitted owing to a 10-cm mass in her left breast. Three years prior, a soybean-size mass was initially discovered without tenderness, nipple overflow, surface skin redness, swelling, elevated local skin temperature, fever or chills, low heat night sweats, chest pain and tightness, or nausea and vomiting. The lesion grew rapidly within 1 month. Physical examination revealed a palpable painless mass in the left breast, which was approximately 10 cm in diameter.

**Figure 1.**
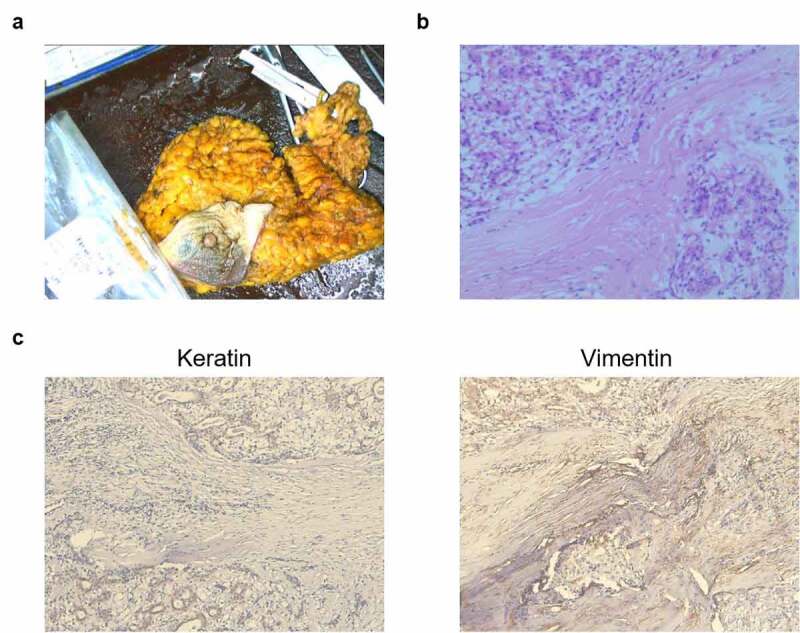
**Representative images of postoperative samples from a 68-year-old Chinese woman with an adenomyoepithelioma (AME)**. (a) An image of AME tissues. (b) A typical image of hematoxylin and eosin-stained postoperative tissues. Scale bar = 100 μm. (c) Immunohistochemistry assay for keratin and vimentin expressions. Scale bar = 100 μm

Pathological examination affirmed the diagnosis of a malignant AME of the breast. Findings of hematoxylin and eosin staining revealed the presence of myoepithelial spindle cell type (Figure 1b), and immunohistochemistry results showed the expression of the epithelial cell marker keratin and the mesenchymal cell marker vimentin (Figure 1c).

### Establishing AME patient‑derived organoids

**Figure 2.**
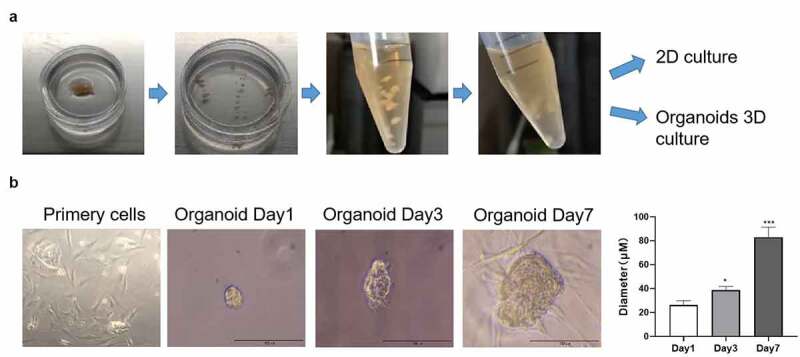
**Images representing the process of culturing organoids from an adenomyoepithelioma (AME) of a patient**. (a) The AME tumor sample was cut into 1-mm^3^ pieces and digested with 2 mg/mL collagenase. The tumor cells were then cultured under 2D and 3D culture systems. (b) The morphology of the cultured tumor cells and organoids at days 1, 3, and 7, as observed under a microscope. The diameter of the organoids was measured at days 1, 3, and 7. Data are presented as mean ± standard deviation. n = 3, *p < 0.05, ***p < 0.001

To establish AME patient-derived organoids, the specimen was collected from the patient. We obtained 1-mm^3^ pieces from the AME sample and digested these using 2 mg/mL collagenase and 1.2 U/mL dispase II (Figure 2a). We obtained tumor cells from the samples for culturing them under two-dimensional (2D) and 3D culture systems (Figure 2b). The tumor cells were spindle-shaped in a 2D cell culture system (Figure 2b). The organoids were cultured well in a 3D-culture system, and we kept a record of the culturing process when the organoids were cultured for 1, 3, and 7 days (Figure 2b). The diameter of organoids was approximately 20, 40, and 80 μm when they were cultured for 1, 3, and 7 days, respectively (Figure 2b). The organoids filled up the cultivation space after they were cultured for 10 days.

### STR profiling of AME organoids revealed a match with the original tissue

**Figure 3.**
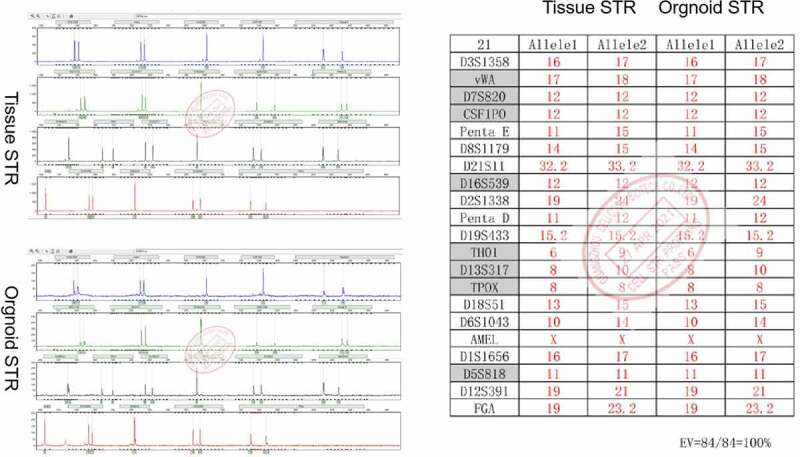
**Short Tandem Repeat (STR) profiles of the adenomyoepithelioma (AME) organoids and the original AME tissue**. The STR profiles revealed a 100% match between the two

To confirm that the tissues and organoids were of the same origin, STR profiling was performed. Our analysis revealed that the STR profiles showed a 100% match between the AME organoids and the original tissue (Figure 3a). The 21 identified loci were D3S1358, vWA, D7S820, CSF1P0, Penta E, D8S1179, D21S11, D16S539, D2S1338, Penta D, D19S433, TH01, D13S317, TPOX, D18S51, D6S1043, AMWL, D1S1656, D5S818, and FGA (Figure 3b).

### Sensitivity of AME organoids to chemotherapeutic drugs

**Figure 4.**
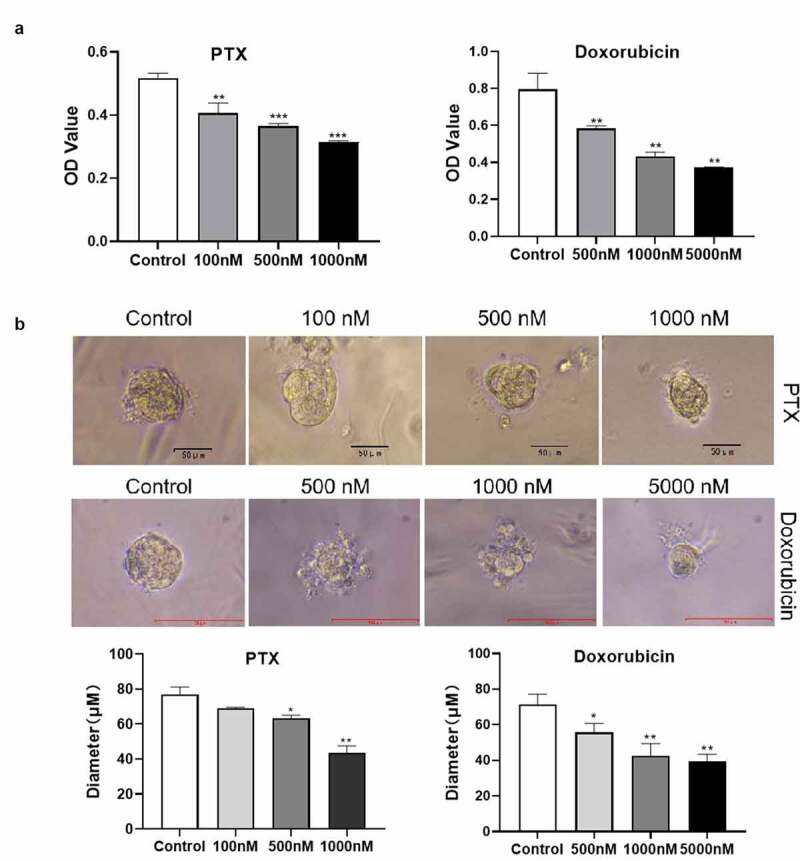
**Sensitivity of primary adenomyoepithelioma (AME) tumor cells and AME organoids to chemotherapeutic drugs**. (a) A representative image showing the viability of primary AME tumor cells, derived from an AME of the left breast of a Chinese woman, after paclitaxel and doxorubicin treatments, which was confirmed using Cell Counting Kit-8 assay. (b) A representative image of organoids treated with paclitaxel and doxorubicin. The diameter of organoids after paclitaxel treatment was measured. Data are presented as mean ± standard deviation. n = 3, *p < 0.05, **p < 0.01

To determine the sensitivity of AME organoids to chemotherapeutic drugs, the organoids were treated with paclitaxel and doxorubicin, which are anticancer drugs that have been widely used in the treatment of breast, ovarian, some head and neck, and lung cancers [20–22]. CCK-8 assay confirmed that paclitaxel and doxorubicin treatments significantly decreased the viability of primary AME tumor cells cultured in a 2D cell culture system (Figure 4a). Treatment with 100 nM paclitaxel had no obvious effect on the diameter of AME organoids. However, the diameter of AME organoids reduced remarkably after treatment with 500 nM and 1000 nM paclitaxel (Figure 4b). Doxorubicin significantly inhibited the growth of organoids at 500 nM, 1000 nM, and 5000 nM. The above results revealed that both primary AME tumor cells and organoids were sensitive to paclitaxel and doxorubicin treatments. However, AME of the breast organoids is less sensitive to paclitaxel than primary culture cells.

## Discussion

AME of the breast is rare and is found in only 1.0% of the cases of breast cancers [23]. However, a small number of AMEs undergo malignant transformation, which may recur locally and have a metastasis rate of 30%–40%, albeit the benign nature of AMEs [24]. AMEs occur more commonly in postmenopausal women [25]. As AMEs are rare, it is impending and necessary to establish biologically and clinically relevant models that will be of significant help in finding effective drugs to improve the therapeutic outcomes. In the present study, an elderly woman was admitted because of a > 10-cm mass in her left breast, which grew quickly within 1 month.

A 3D culture system, a novel model system that mimics the rich *in vivo* microenvironment and complex processes in which cells grow, is superior to the traditional 2D cell culture system, which has been extensively used in previous studies [26–28]. Thus, a 3D culture system offers great promise for the preclinical evaluation of drug efficacy and associated risks. To date, several types of 3D culture systems have been established, not only from single cell-type static 3D culture systems to cell coculture 3D culture systems but also from simple spheroids to organs-on-chips [29–31]. At present, some organ models, including the gut or blood–brain barrier, lung, liver, and kidney, have been established to investigate drug pharmacology [11,32–34]. However, there are still no reports of organoids derived from patients with AME that have been established for nonclinical testing. In the present study, we successfully established an AME organoid culture system from original tumor samples. The STR profiles showed a 100% match between the AME organoids and the original tissue. In addition, our analysis revealed that both primary AME tumor cells and organoids were sensitive to paclitaxel and doxorubicin treatments. However, more study need to confirm the genetic signature of organoid compared to tumor from the frozen sample using mRNA transcriptome sequencing.

Compared with a 2D cell culture system, such as Wang’s and Yao’s researches used [35,36], a 3D organoid culture system mimics the *in situ* tissue more closely. Thus, tumor-derived organoids from AME of the breast are a promising model for the clinical assessment of treatment regimens, evaluation of new drugs, and research on drug targets. However, organoids are cultured *in vitro*, thereby lacking immune responses, hypoxia, and vascular microcirculation, which are present in *in vivo* conditions. Therefore, it is necessary to establish organoids and organoid chip models that can more simulate the *in vivo* environment more accurately. In future, we aim to coculture AME organoids with immune cells and vascular endothelial cells to establish a model that can simulate the *in vivo* environment more accurately.

## Conclusion

An AME organoid model was established from an AME of the breast discovered in a 68-year-old Chinese woman. AME organoids can be effectively used for exploring clinicopathological and genomic characteristics of patients with AME.

## Highlights

1. DNA signature of the AME of the breast organoids matched the original tumor specimen.
2. AME can be used to establish cancer organoid models.
3. AME organoids were sensitive to the chemotherapy drugs paclitaxel and doxorubicin.

## Data statement

The data can be provided upon reasonable request to the corresponding author.
